# Can mHealth improve migrant wellness during public health emergencies? A community-engaged qualitative study during the COVID-19 pandemic

**DOI:** 10.1186/s44247-026-00253-0

**Published:** 2026-03-16

**Authors:** Chad Abresch, Michelle Warren, Ellen Kerns, Alice Sato, Gleb Haynatzki, Jonathan Figliomeni, Fernando Sanchez, Gisela Marfileno, Lisvey Rivera, M. Jana Broadhurst, Russell J. McCulloh

**Affiliations:** 1 Department of Health Promotion, College of Public Health, University of Nebraska Medical Center, Omaha, NE USA; 2 Department of Modern Languages, College of Arts & Sciences, University of Nebraska Kearney, Kearney, NE USA; 3 Department of Pediatrics, College of Medicine, University of Nebraska Medical Center, Omaha, NE USA; 4 Department of Biostatistics, College of Public Health, University of Nebraska Medical Center, Omaha, NE USA; 5 Child Health Research Institute, University of Nebraska Medical Center, Omaha, NE USA; 6 Central District Health Department, Grand Island, NE USA; 7 Child Health Research Institute, University of Nebraska Medical Center, Kearney, NE USA; 8 Nebraska Migrant Education Program, Kearney, NE USA; 9 Department of Pathology & Microbiology, College of Medicine, University of Nebraska Medical Center, Omaha, NE USA

**Keywords:** Mobile health, Digital health, Migrant populations, COVID-19, Community navigators

## Abstract

**Background:**

The COVID-19 pandemic exposed significant healthcare access disparities for migrant communities, with infection rates up to three times higher than the general population. While mobile health technology offers potential solutions for reaching vulnerable populations during public health emergencies, implementation barriers persist. This study evaluates how mobile health interventions can effectively serve migrant communities through analysis of a digital health program implemented in rural Nebraska.

**Results:**

Analysis of semi-structured interviews with migrant family members between February 2022 and June 2024 revealed three key elements driving program success: culturally congruent support through community navigators, immediate access to testing and results, and program adaptability to meet broader community needs. Community navigators proved essential, expanding beyond technical support to address social needs ranging from food insecurity to domestic issues. The combination of digital tools with human support enabled families to make timely decisions about work and school attendance while accessing crucial social services.

**Conclusions:**

Digital health interventions can effectively serve migrant communities when designed with cultural sensitivity and supported by community navigators. While public health systems should prioritize technological infrastructure for emergency response, success requires concurrent investment in culturally responsive human support systems. The integration of digital tools with adaptable community navigation provides a model for reaching vulnerable populations during future public health emergencies.

**Supplementary Information:**

The online version contains supplementary material available at 10.1186/s44247-026-00253-0.

## Background

The COVID-19 pandemic claimed over 1.1 million lives in the United States [1], bringing profound personal loss and exposing significant inequities in healthcare access and outcomes [2]. Migrant communities experienced disproportionate impacts, with infection rates up to 3 times higher than the general population and significantly lower access to testing and preventive services [3, 4]. Structural inequities in healthcare access and delivery, including vaccine access, led to disproportionate COVID-19 mortality rates in migrant agricultural settings [5, 6].

Migrant communities faced compound challenges during the pandemic, stemming from both occupational exposure and systemic barriers [7, 8]. As essential workers in agriculture, food processing, and other critical industries, migrants experienced heightened infection risks while often lacking basic health protections. These occupational hazards intersected with structural barriers including limited healthcare access, language differences, and economic instability [9]. Challenges were further exacerbated by inadequate public health infrastructure in rural areas, where many migrant families live and work [10, 11].

Public health systems struggled to effectively connect to migrant populations during the pandemic [12]. Traditional outreach methods proved insufficient, while social distancing requirements further impeded community engagement [13]. Healthcare providers and public health departments reported challenges in maintaining consistent communication with migrant communities, coordinating testing and vaccination efforts, and providing culturally appropriate health information [14, 15].

Mobile health (mHealth) technologies emerged as a potential solution for addressing such challenges [16–18]. These digital tools can facilitate bi-directional information sharing, enhance care access, and support health-protective behaviors [19]. During public health emergencies, mHealth applications have demonstrated effectiveness in symptom monitoring, exposure tracking, and health education delivery [20]. However, technology, privacy, and cultural barriers challenge the implementation of mHealth interventions among migrant communities [19, 21].

Our study applies the Consolidated Framework for Implementation Research (CFIR) [22] to evaluate how intervention characteristics, organizational factors, external context, individual characteristics, and implementation processes influence mHealth program success in migrant communities. As a theoretical foundation for understanding barriers and facilitators to program adoption in resource-limited settings, CFIR guides our analysis of how effectively digital health solutions were implemented to support vulnerable populations during public health emergencies.

Through qualitative investigation of an mHealth intervention serving migrant families in rural Nebraska as part of the NIH RADx-UP initiative [23], this study aims to inform the development of more equitable and effective digital health solutions during future public health emergencies.

## Methods

### Study design and setting

We conducted a community-engaged qualitative study to evaluate the Mobile Health for Migrant Health (mHealth-4-Mhealth) intervention, implemented in partnership with the Nebraska Migrant Education Program (MEP). The MEP is a federally funded program administered by the U.S. Department of Education that provides educational and supportive services to children of migrant agricultural workers. MEP staff, including community navigators, serve as trusted liaisons between migrant families and local services, making the program a natural partner for health-related outreach in these communities [24]. The study took place from February 2022 to June 2024 as part of a statewide program, with study participants drawn from three rural Nebraska counties (Adams, Buffalo, and Hall) with consistent COVID-19 transmission throughout the pandemic [25]. These counties have substantial migrant agricultural worker populations. Hispanic or Latino residents make up approximately 30% of Hall County’s population, 12% of Adams County’s population, and 10% of Buffalo County’s population, according to U.S. Census data [26]. Hall County’s Hispanic population is notably higher than the Nebraska state average of approximately 12%, while Adams and Buffalo counties are near and just below the state average, respectively. All three counties have documented migrant and seasonal agricultural worker populations, with Hispanic residents comprising a substantial share of the agricultural labor force [27]. Initial program implementation and quantitative outcomes were reported by McCulloh et al. [20] Our study reports on extended program engagement utilizing qualitative data collection through June 2024.

The mHealth-4-Mhealth intervention comprised three core components: (1) a bilingual mobile application facilitating daily symptom screening and infection risk assessment, (2) coordinated distribution of at-home SARS-CoV-2 antigen test kits, and (3) integrated community navigation services (i.e., community navigators employed directly by our research team). The mobile application provided guidance on healthcare access and work/school attendance decisions, while weekly screenings identified social challenges such as housing instability and financial strain. The mHealth app was initially designed to provide information to community navigators to connect participants with needed services following established models of community health worker engagement.

### Study population

Participants were adult members of households with children who met federal MEP eligibility criteria (children under 22 years old who moved within the last 36 months with a parent or guardian seeking temporary or seasonal agricultural work). We used two inclusion criteria for interview selection: (1) a positive COVID-19 symptom screen recommending testing, and (2) indication of whether they were willing to conduct SARS-CoV-2 at-home testing. By June 2024, the program had expanded to include 99 households comprising 407 participants. The predominance of Spanish-speaking Latina mothers in our sample (100%) reflects the typical family structure within MEP-eligible households, where mothers commonly serve as primary caregivers and health decision-makers. This pattern is consistent with established research on Latinx immigrant family structures and gendered caregiving roles in migrant agricultural communities [21, 28].

### Recruitment and data collection

The three counties involved in this study were purposively selected based on their presence of migrant agricultural workers, consistent COVID-19 transmission rates, and existing partnerships with MEP staff. To select individual participants, our study employed two sampling approaches. First, community navigators used purposive sampling to identify participants who had experienced barriers to accessing essential needs during the pandemic, including housing instability and difficulties accessing healthcare due to documentation status and other socioeconomic stressors. Second, we reviewed monthly contact lists from our project database to recruit participants who had received app-based recommendations for COVID-19 testing, selecting an equal distribution of individuals who did and did not follow through with testing. This dual approach allowed us to examine both specific challenges faced by vulnerable community members and patterns of engagement with the intervention.

A team of bilingual, bicultural researchers conducted semi-structured interviews in participants’ preferred language (Spanish) [29]. All team members completed training in qualitative interviewing, data security protocols, cultural competency, and review of the interview guide (Supplemental File). Interviews averaged approximately 25 min, were conducted via teleconference, and were recorded with consent. Interview participants received no additional compensation beyond the study participation remuneration of $20 every two weeks, provided to all enrolled households as compensation for their ongoing engagement with the mHealth-4-Mhealth program, including completing regular symptom screenings and surveys.

### Analysis

**Fig. 1:**
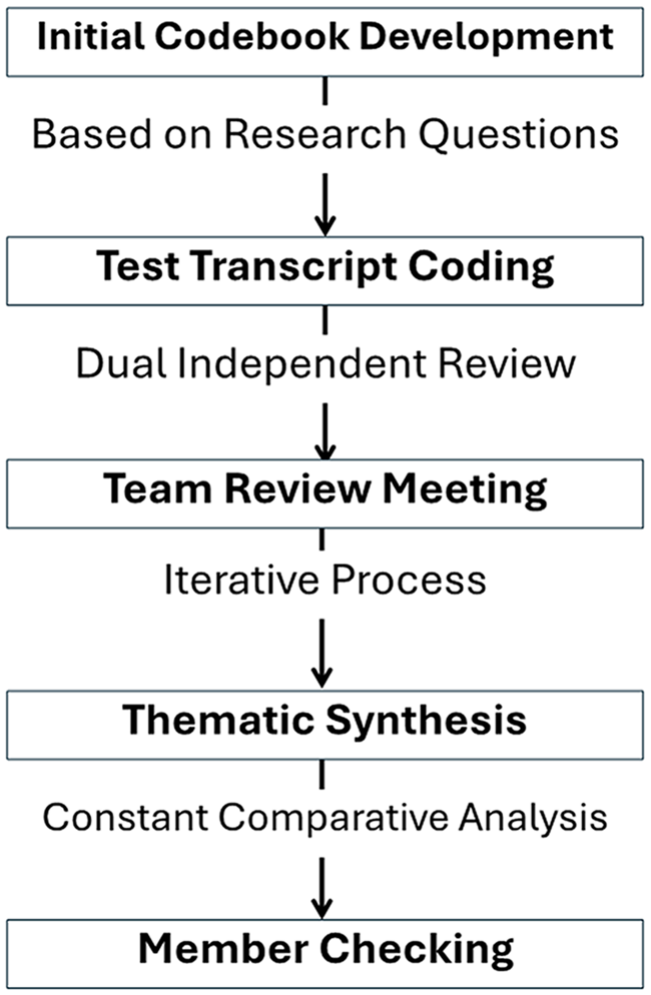
Qualitative analysis workflow

We employed rigorous thematic analysis following the framework described by Braun and Clarke^30^ which involves six phases: familiarization with the data, generating initial codes, searching for themes, reviewing themes, defining and naming themes, and producing the report. Our coding approach used a hybrid strategy combining deductive codes derived from the CFIR framework domains with inductive codes that emerged directly from participant narratives. The four-member coding team independently reviewed each transcript, developed initial codes, and then met to compare coding, discuss discrepancies, and reach consensus on the final codebook through iterative discussion. Thematic saturation was assessed throughout the data collection process; after multiple interviews with no new themes emerging across consecutive interviews, the investigative team jointly confirmed saturation had been reached [30]. Saturation was the sole criterion for concluding data collection; no additional interviews were conducted because new themes had ceased to emerge, not due to participant attrition or logistical constraints. Remaining enrolled households continued to participate in the broader mHealth-4-Mhealth program activities but were not contacted for qualitative interviews. Interviews were professionally transcribed verbatim and translated into English by a native Spanish speaker, approved by our institutional review board. Four team members (CA, MW, JF, GM) collaboratively conducted systematic coding using Dedoose software. Figure 1 outlines our qualitative analysis process.

## Results

The results are organized according to the five domains of the Consolidated Framework for Implementation Research (CFIR): Intervention Characteristics, Outer Setting, Inner Setting, Characteristics of Individuals, and Implementation Processes. Themes identified during analysis are presented within each domain, reflecting how participants’ experiences aligned with these implementation constructs. Our analysis of interviews with 10 migrant family members (by which point we had reached thematic saturation [31] with no new themes emerging in the final three interviews) revealed how multiple factors influenced the implementation of the mHealth-4-Mhealth intervention during the COVID-19 pandemic. Characteristics of participants are summarized in Table 1.

**Table 1:** Demographic characteristics of interview participants (*n* = 10), mobile health for migrant health program, Rural Nebraska, 2022–2024

Characteristic	Value
Age, median (IQR), y	40 (37.5, 44.5)
Sex, No. (%)	
Female	10
Male	0
Race, No. (%)	
White	5
Black	0
More than one race	0
Other	5
Ethnicity, No. (%)	
Hispanic/Latino	10
Non-Hispanic/Latino	0
Preferred Language, No. (%)	
Spanish	10
English	0
Bilingual (Spanish/English)	0
Other	0
RUCA Classification, No. (%)	
Metropolitan	0
Micropolitan	10
Small Rural	0

### Intervention characteristics

The mHealth-4-Mhealth intervention’s design features significantly influenced its adoption and effectiveness. Participants consistently highlighted three key characteristics: culturally congruent support, ease of use, and immediate access to testing and results. The presence of bilingual, culturally congruent staff proved crucial for family engagement and was mentioned 9 times by participants. One participant described the value of personalized support: “I had questions about the app at the beginning, and [your community navigator] took the time to come to my house and explained, ‘Do this like this. And this like this. And like this.’ It’s been easier for me since then” (Participant D-1).

The straightforward symptom screening process facilitated regular use and promoted sustained engagement. Participants emphasized this point 11 times with one participant noting: “It’s very easy to do it because there are only two, three questions and that’s it. I can answer it just on my phone, and that’s it. It’s simple” (Participant A-1). Conversely, the absence of push notifications limited participant engagement. This same participant noted: “Honestly, I forget it. It doesn’t notify you, so you think, ‘Ay, did I answer it or not?’ [laughs]. So, I do it again” (Participant A-1).

The availability of at-home testing was viewed positively. Participants valued the convenience and immediacy of results compared to traditional testing approaches. One mother explained: “Before, we had to schedule an appointment to get tested, and we got the results three days later. Now, I can also test my young daughter, and it’s easier for my family” (Participant D-1). The ability to quickly confirm COVID-19 status provided participants with greater confidence in their health decisions. As one participant noted: “I felt more confident after testing. And I thought, ‘Well, if I’ve got COVID, it will show it; or if I haven’t, it will show it too’” (Participant G-1). This combination of convenient testing access and immediate results helped families make timely decisions about work, school, and family activities.

### Outer setting

External factors significantly shaped how families engaged with the mHealth-4-Mhealth intervention, particularly employment disruptions, financial impacts, and community-level COVID-19 responses. Employment challenges emerged as a prominent theme, mentioned 16 times by participants, with workplace testing policies and exposure protocols creating uncertainty. One participant described this disruption: “My husband had to leave work for ten days. Actually, he got tested, but they lost the results… Since he commuted with other people to work, he was in contact [with someone who tested positive], so, automatically he was sent home” (Participant A-1).

The economic consequences of these employment disruptions affected many families, with 10 participants reporting financial strain. As one noted: “It also changed economically because we didn’t work the same hours, my husband worked less hours because, as you know, sometimes people were sent home” (Participant B-1). The program’s research compensation helped offset some of these financial challenges. One participant emphasized this benefit: “We also get support from you guys, right? That’s very important, you know, it’s an extra for our household… Yes! … It’s a huge help” (Participant I-4).

Community-level COVID-19 responses, particularly in schools, created additional complexity for families managing health decisions. Unpredictable closures and decontamination protocols affected daily routines and exacerbated ongoing pandemic impacts. One participant explained: “A kid got sick two weeks ago and they cancelled classes, right? They said, ‘Well, you have to pick up your kids. There won’t be school tomorrow because we are going to disinfect the classrooms.‘” (Participant D-1). The uncertainties and disruptions in the external context seemed to enhance program participation. For example, when the same interviewee was asked if the program helped, she said yes and added, “I have control of my family’s health” (Participant D-1).

### Inner setting

While the CFIR domain of “Inner Setting” traditionally examines organizational contexts, our analysis revealed how household environments and family dynamics significantly influenced program implementation. Although this domain typically focuses on the implementing organization, we adapted it to examine the household as the primary setting where participants engaged with the intervention. This adaptation was necessary because the mHealth-4-Mhealth intervention was delivered directly to families in their homes rather than through a traditional healthcare organization, making household dynamics a critical factor in implementation success. Three key aspects of the home setting emerged as crucial: establishment of strict health protocols, adaptation to remote schooling demands, and management of isolation requirements.

Families developed elaborate routines to prevent COVID-19 transmission. One participant described their comprehensive approach: “It looks a bit exaggerated; but my kids and I take off our clothes and wash them right away when we get home… when we go to crowded places, we try not to go, but if we have to go… when we get home, we use Lysol; I have one at the door and in the car as well. We take off our shoes and leave them outside… My daughter says I overreacted, but it has worked pretty well” (Participant D-1).

Remote schooling created significant challenges within the home environment, mentioned 12 times by participants. In addition to the demands of having children home during the day, participants expressed concern about reduced learning, “They would send us fliers, so the kids would know where to go to study. It was a change because I think they didn’t learn as much as going to school, you know, it’s not the same. They only took one class; it wasn’t a lot” (Participant I-4).

Perhaps most disruptive to the home environment were isolation requirements when family members contracted COVID-19. Seven participants described dramatic changes to family routines during quarantine periods. One participant, living in a shelter, recounted: “My daughter and I got COVID… They had to isolate us to quarantine, right? They locked us down for twenty days. They would bring us food and leave it at the door. We couldn’t go anywhere; just to the bathroom” (Participant J-2).

In sum, the “inner setting” examples revealed that our participants adhered to the COVID-19 public health precautions and to the program. This may have stemmed from their vulnerable place in society as migrant workers and families. One participant summarized the situation as follows: “Thank God, everything is better. Maybe everything is more controlled. We have now available home tests. We have everything available, right? You provide us with at-home tests kits to test ourselves. So, we have more access to that, it’s easier to find out if we have COVID or not. Back then, we would call our job and say, ‘I’m sick and I have these symptoms,’ and they would say, ‘You can’t come’” (Participant D-1).

### Characteristics of individuals

Individual perceptions, experiences, and emotional responses to COVID-19 significantly shaped how participants engaged with the mHealth intervention. Four key themes emerged: personal realization of COVID’s reality, fear of infection, trauma and loss, and vaccine decision-making.

For some participants, personal experience was necessary to fully grasp COVID-19’s severity. As one reflected: “I experienced it in my family, I mean, it wasn’t a joke; it was real, we shouldn’t be joking about it and saying that it doesn’t exist” (Participant C-1). This recognition of COVID’s reality often catalyzed program engagement and adherence to health protocols.

Fear of COVID-19 emerged as a dominant theme, mentioned 18 times across interviews. Participants expressed particular concern about household transmission. One mother explained: “I was worried in case someone got infected, and that that person would infect the rest at home. Since my kids are in practice and they are in contact with other groups, my first worry was to find out if we were infected to avoid spreading it with friends, and here at home” (Participant B-1).

Personal experiences of loss and trauma, mentioned by 9 participants, profoundly influenced health behaviors and program participation. One participant shared her family’s transformation after losing a loved one: “It will be the second anniversary of my father-in-law’s death, he got COVID and passed away in México. It was really shocking because he was a very healthy person. We never imagined, actually, we didn’t believe in… my husband was one who didn’t believe in the illness [laughs]. We didn’t believe it was true. And, unfortunately, we experienced it” (Participant A-1). This loss led to heightened health precautions, as she later explained: “When the kids in school stopped wearing masks, I asked my kids to still wear them, and they would say to me, ‘Mommy, we are the only ones who wear masks in the classroom’… but again, for the reason of my father-in-law and all that… I said, ‘No, wear them because we don’t know how things are.‘”.

Notably, while most participants readily accepted all COVID-19 prevention measures, vaccine hesitancy emerged in one case. This participant, while fully engaged with the mHealth program’s testing and screening protocols, expressed uncertainty about vaccination: “We haven’t been vaccinated against COVID in my household. My husband and I have talked about it many times. We even made an appointment one day, but we didn’t get vaccinated because, Ay! I don’t know… I’ve seen they get really sick when they get the vaccine… I don’t know how to explain it” (Participant E-1). This selective engagement with prevention measures illustrates how individuals could maintain strong program participation while holding reservations about specific COVID-19 interventions.

### Implementation processes

The implementation of mHealth-4-Mhealth revealed both the challenges and opportunities of delivering digital health interventions to migrant communities. Our analysis identified four key aspects of the implementation process: initial engagement motivations, implementation challenges, program adaptations, and evolving community support roles.

Program engagement was strong, mentioned 18 times by participants, driven largely by parents seeking ways to protect their families while navigating unfamiliar social and healthcare systems. As one participant explained: “The first time I used it [at-home test kit] for my little girl, she had a sore throat. Then the other time for my husband because he had a fever for two days… I think it helps all the Hispanic families that live here and are part of the migrant program, because not all of them have insurance or are able go to the doctor, you know, it’s expensive” (Participant E-1).

Implementation challenges were noted 11 times by participants and ranged from technical issues to usability concerns. Some participants struggled with basic app functionality: “The only option was to use the app, but it’s one of the most difficult things for me. I don’t understand them; for me it’s very difficult to use apps” (Participant J-2). As reported above, the absence of push notifications with the mHealth app emerged as a consistent barrier to regular engagement.

In response to these challenges, the program evolved to include more intensive personal support through culturally congruent community navigators. This adaptation proved crucial for sustained engagement, as one participant described: “Honestly, I didn’t know how to use it at the beginning, until you took the time to show me how. I have used it since then… I do one one day, and the other one, the next day, so, I don’t forget it. If my kids get sick, I use a test kit, right? Otherwise, I use the app the way you showed me” (Participant D-1). When automated notifications weren’t possible, navigators provided manual reminders through WhatsApp messages, which participants found “very helpful” (Participant D-1).

Perhaps most significantly, the implementation process revealed how community navigators became essential bridges between the digital intervention and participants’ complex lives. Navigators addressed a broader range of needs than anticipated, from food insecurity to transportation to domestic issues. This expanded role, while taxing program scope and resources, deepened community trust. As one participant noted: “I like that you are interested in the community, willing to help” (Participant C-1). The intervention’s value was particularly evident for families managing transnational moves, as illustrated by one participant who contrasted their experience in Guatemala with the program’s support: “Your help has been very important for me and my kids… I didn’t have a job. I didn’t have any food or help. I was in Guatemala… [but now] I’ve got your support, and that has been helping me. For me, it’s easier being here than in Guatemala” (Participant J-2).

## Discussion

Our study demonstrates how digital health interventions can effectively serve migrant communities during public health emergencies when designed with cultural and contextual sensitivity. Three key elements—culturally congruent support, immediate access to testing and results, and the adaptability of community navigators—drove program success and provided guidance for implementing similar interventions. Our findings demonstrate how documented technology barriers for minority populations [32, 33] can be overcome through inclusive and adaptive design elements and intentional human interaction. Cultural sensitivity was embedded in the intervention through multiple mechanisms: the bilingual mobile application was developed with input from community navigators who understood the cultural context of migrant families, all participant-facing staff were bilingual and bicultural, and the program maintained flexibility to adapt to the cultural norms and preferences of participants. Community navigators served as cultural brokers, ensuring that health information was communicated in culturally appropriate ways and that the digital tools aligned with participants’ communication preferences and daily routines.

The role of community navigators emerged as essential, evolving beyond traditional health worker duties [34]. While the mHealth platform was effective, it naturally had technical constraints. Navigator adaptability transformed these limitations into an opportunity for sustained and meaningful engagement with the migrant community. Navigators compensated for technological limitations and expanded their roles to address needs ranging from food insecurity to domestic issues. This affirms recent findings on community health worker effectiveness during COVID-19 [35–37] and demonstrates how human support remains crucial in digital health implementation.

Immediate access to testing and results proved transformative for families navigating complex work and school environments. At-home testing empowered participants to make timely decisions about work/school attendance and family interactions. For migrant families, who often lack paid sick leave and face employment insecurity [38], the ability to quickly confirm COVID-19 status became a crucial tool for protecting both health and livelihood.

The transnational nature of participants’ movement between the U.S. and their home countries requires flexible support systems. Recent research has documented how transnational migration patterns complicate healthcare delivery through disruptions in continuity of care, loss of established provider relationships, challenges in transferring medical records across borders, and difficulties maintaining consistent preventive health behaviors during periods of transition [39]. Our findings suggest that the combination of rapid testing access and adaptable navigator support helped bridge these gaps. The mHealth platform’s portability and the navigators’ ability to maintain relationships with families across moves provided a degree of continuity that traditional healthcare systems often cannot offer to mobile populations.

Our results also highlight how external contextual factors—particularly employment conditions and school policies—influence mHealth adoption in migrant communities. The intervention’s effectiveness stemmed from its ability to help families navigate these systemic challenges by adding human support, corroborating recent calls for interventions that address social drivers of health [40]. The program’s evolution to meet broader community needs demonstrates the importance of flexible implementation during public health emergencies.

Our findings have significant implications for public health practice, policy, and infrastructure. For practice, mHealth interventions focused on vulnerable populations must explicitly plan for technological limitations by incorporating resources for human support. The success of our navigators demonstrates how critical human elements are in digital health implementation.

The integration of at-home testing with digital platforms provides a model for future emergency response. Our findings show how immediate access to actionable health information enables vulnerable populations to better navigate complex environments. Public health systems should prioritize rapid-response, particularly for communities facing structural barriers to healthcare access.

At a policy level, our findings highlight critical gaps in health protection for migrant workers. While digital health interventions can help mitigate immediate challenges, sustainable public health improvements require addressing underlying inequities, including health insurance and sick leave. Policymakers should consider how workforce protections intersect with public health emergency response, particularly for vulnerable populations.

### Limitations

Several limitations warrant mention. While our study demonstrated the importance of human workarounds for technological limitations, we were unable to test whether building an app with more efficiency would reduce the burden on navigators. Additionally, since the study coincided with evolving COVID-19 policies, it is difficult to isolate intervention successes from broader contextual changes. Furthermore, because qualitative interviews were conducted between February 2022 and June 2024 while the mHealth-4-Mhealth program began earlier, some participants were recalling experiences that occurred months to over a year prior. This temporal gap introduces the possibility of recall bias, as participants’ recollections may have been shaped by subsequent experiences or evolving perceptions of the pandemic. Additionally, participants received financial incentives ($20 every two weeks) during a period of significant economic hardship, which may have influenced their willingness to participate and the positivity of their responses. The purposive and database-driven sampling strategy, while appropriate for our research questions, introduces potential selection bias inherent in most qualitative research. Our sample was drawn from participants already enrolled in the MEP and the mHealth-4-Mhealth program, which may not capture the experiences of migrant families who were unreachable or chose not to engage with either program. To mitigate this, we employed a dual sampling approach that included both navigator-identified participants facing specific barriers and database-selected participants representing varied levels of engagement with testing recommendations. Finally, the homogeneity of our sample—exclusively Spanish-speaking Latina mothers—while reflective of the MEP-eligible population in these counties, limits the transferability of findings to other migrant subpopulations or geographic contexts.

## Conclusion

This study demonstrates that digital health interventions can effectively serve migrant communities during public health emergencies when designed with cultural sensitivity and supported by community navigators. By combining technological tools with adaptable human support, the intervention successfully helped migrant families navigate complex health decisions while addressing broader social needs. The findings highlight how immediate access to testing coupled with culturally congruent support can empower vulnerable populations to protect both their health and livelihoods during crises. As public health systems prepare for future emergencies, our results emphasize the importance of building and maintaining both technological infrastructure and culturally responsive human support networks. These parallel investments in digital and human capital are essential for creating more equitable and effective emergency response systems that can reach and serve all communities, particularly those facing multiple barriers to healthcare access.

## Supplementary Information

Below is the link to the electronic supplementary material.

Supplementary Material 1

## Data Availability

The datasets used and analyzed during the current study are available from the corresponding author on reasonable request and with participant privacy held paramount.
