# Supplementary material for: Can mHealth improve migrant wellness during public health emergencies? A community-engaged qualitative study during the COVID-19 pandemic
Source: BMC Digit Health. 2026 Mar 16;4(1):16. doi: 10.1186/s44247-026-00253-0 (PMC12992342; doi:10.1186/s44247-026-00253-0)
Supplement: Supplementary file 1 — Supplementary Material 1 [file 44247_2026_253_MOESM1_ESM.pdf]

Qualitative Data Collection

Relevant Research Questions

3. Qualitative investigation into program reluctance

- What socioeconomic challenges negatively impact the ability and willingness of migrant families to safeguard their health and wellbeing during COVID-19?
- What changes (e.g., public aid, legal protections, community programs, etc.) do migrant families believe are necessary to enhance their ability and willingness to safeguard their health and wellbeing during COVID-19 and similar future events?

Qualitative Research Participants

Participants will be adult household members (e.g., parents, guardians, etc.) of children eligible, enrolled, or who previously participated in MEP programs in Nebraska's Adams, Buffalo, and Hall counties who: 1) complete the COVID-19 symptom screener with indication of infection risk; and, 2) respond that they (a) are or (b) are not willing to perform at-home SARS-CoV-2 testing. Additionally, we will [consider] interviewing additional households that have not used the app (depending on availability of interviewees) to determine why they have not used the app/program. And/or interview households that test, but did not use the app.

Qualitative Research Procedures

- **Identification**. Monthly, Kerns will send 10 (5 from each group) randomly sampled households' contact info to MEP study staff.
- **Interviewing**. Study staff will contact households to conduct recorded interviews (target: 1-2 interviews monthly for both groups). Face-to-face and virtual interviews will be used (Zoom may have both English and Spanish transcription).
- **Translation and Transcription**. In development. Currently translating this guide into Spanish. Need to identify transcription service or technology. Considering delayed translation in favor of transcription and analysis in Spanish (per van Nes et al., 2010) with parallel quote presentation (i.e., Spanish and English).
- **Coding**. Abresch, Warren, Sanchez, Marfileno will code for themes on an ongoing basis to identify potential shifts in themes. Constant comparative analysis will be applied to identify similarities and differences between the two groups.

Interview Guide

Interviewer Preparation

Before beginning an interview, briefly reflect/center on our Interviewing Core Values.

- **Care.** We genuinely care about the people we interview and want them to feel that care. The best interviews can be cathartic.
- **Conversation.** We want to engage in conversation with our interviewees, not just get their "answers" (otherwise we'd use a survey).

- **Character.** Our interviewees are the main characters in their STORIES and we want to hear their stories. Draw out their stories with “What did you do?” and “What happened next?”.
- **Quality.** We hit all the fundamentals every time (we show up early, we’re prepared and organized, we always get consent, the recording is done correctly, etc.).

Introduction (5 mins)

Hi ________, my name is _________. Thank you for taking time to talk with me today. I want to talk with you so I can learn what things in your life make it hard for you and your family to stay healthy during the COVID-19 pandemic.

I'm going to start by reading a few short statements about the study and asking if you are willing to talk with me today. Does that sound okay?

- We ask that you silence your cell phones if possible.
- There are no right or wrong answers, only different points of view. We are as interested in negative comments as positive ones.
- We will record this session because we don't want to miss any of the conversation.
- The study team and I will never use your name or identify you in our reports. Everything said here today will be kept strictly confidential.
- You are free to keep your camera on or turn it off. If you start with your camera on and you change your mind, you are may to turn it off at any time. If you do not know how to turn off the camera, please ask us and we can help you.

Are you ready to begin?

Interview (20 mins)

Domain	Interview Question	Possible Probes
Module 1: Household Contextual Factors [USE WITH EVERYONE]	First, I'd like to ask you how life has been for your household during COVID.<br><br>1. Can you start by telling me about **what changed** for your household because of COVID?<br><br>2. What do you think has been the **hardest thing** about COVID for you and your household?	Ensure both school and work comments emerge. If not, probe with:<br>- Thinking about school for the kids in your household, did COVID **make things harder?** How so? Is it still harder?<br>- Thinking about work…
Module 2: Household COVID-19 Mitigation Participation [USE WITH EVERYONE]	I'd like to ask you now about what you and your household did, and still do, to stay safe from COVID.<br><br>1. First, experts asked us to do several things during COVID like social distancing, wearing masks, and doing school or work from home when possible. Did your household do those things?	Probe for barriers to following mitigation recommendations:<br>- Why didn't you…?<br>- What prevented you from…?<br><br>Ensure school and work again.

<table>
<tr><td></td><td>a. All of them?
b. Are you still doing some/all of them?

2. Was it ever hard for your household to follow those COVID safety recommendations?

a. What made it hard?

3. Can you tell me about a time when you or a member of your household wanted to follow COVID safety recommendations, but you didn’t or weren’t able to?</td><td></td></tr>
<tr><td>Module 3: Testing Decision [USE ONLY FOR INTERVIEWS SENT BY EL]</td><td>[For interviewees who took the test when prompted by the app]

In this last section, I want to ask you why you were willing to do the home test for COVID that the app asked you to take.

1. To start: when the app asked you to take the test, what were your thoughts? And, how did it make you feel?

2. What were the main reasons you decided to take the test?

[For interviewees who did not take the test when prompted by the app]

In this last section, I want to ask you why you did not take the home test for COVID that the app asked you to take.

1. To start: when the app asked you to take the test, what were your thoughts? And, how did it make you feel?

2. What were the main reasons you decided to not take the test?

3. What would make it easier for you to take the test?</td><td></td></tr>
<tr><td>Module 4: Program Satisfaction [USE WITH EVERYONE.</td><td>Our program provides your household with an app to track your symptoms and tell you if you need to test for COVID-19. If you do need to test, we have provided you with at-home test kits, which you use and mail back to us.</td><td>Probe for difficulties using the program.

Probe for why they do not use the app.</td></tr>
</table>

SKIP IF TIME IS A FACTOR ON INTERVIEWS FROM EL]	1. How much of this program have you participated in so far? [Use the following sub-questions as necessary]<br>a. Have you enrolled in app?<br>b. How many times have you used the app?<br>c. If you have used the app, have you ever been told to test?<br>d. If so, have you tested?<br>e. Did you hear back from us?<br>f. What other action did you take?<br>2. Can you tell us what you LIKE about this project?<br>3. Great, can you now tell us about what you DO NOT LIKE about this project?	
Conclusion	To wrap up, is there anything we haven't talked about that concerns you or makes it difficult for you and household to get tested and stay safe from COVID?	
